# Supplementary material for: Biotic and abiotic factors affecting Atlantic ghost crab (Ocypode quadrata) spatiotemporal activity at an important shorebird nesting site in Virginia
Source: PLoS One. 2024 Aug 13;19(8):e0307821. doi: 10.1371/journal.pone.0307821 (PMC11321587; doi:10.1371/journal.pone.0307821)
Supplement: S1 Table — Fixed effects varied between models, based on a priori hypotheses about factors thought to affect ghost crab activity. The use of ‘+’ indicates additive terms in a multivariable model, and the use of ‘×’ indicates an interaction between two terms. All models include a random effect for sampling point. Additionally, all models include a zero-inflated component for sampling date. The response variable is ghost crab burrow abundance. (DOCX) [file pone.0307821.s001.docx]

| ***A priori* hypotheses** | **Conditional model component** |
| --- | --- |
| Burrow abundance is explained by date, as ghost crabs are expected to be more active and construct more burrows on the landscape later in the summer during their recruitment period. | (1) Date |
| Burrow abundance is explained by temperature, as ghost crabs are ectotherms that are most likely to be active and construct burrows when air temperatures are optimal. | (2) Temperature |
| Burrow abundance is explained by shell cover, as shells provide surface armoring to the substrate which may affect the ability for ghost crabs to construct burrows. | (3) Shell cover |
| Burrow abundance is explained by habitat, as ghost crabs will construct the most burrows in habitats with minimal vegetation that may obstruct burrowing, such as beaches. | (4) Habitat |
| Burrow abundance is explained by point type, as ghost crabs are expected to be most active around shorebird nests, which offer a prey source, relative to random sampling locations. | (5) Point type |
| Burrow abundance is explained by temperature, but the effect of temperature on burrow abundance depends on the date. | (6) Temperature + date + temperature × date |
| Burrow abundance is explained by whether a nest is present, but the effect of nest presence on burrow abundance depends on the level of shell cover. | (7) Point type + shell cover + point type × shell cover |
| Burrow abundance depends on shell cover and date. | (8) Shell cover + date |
| Burrow abundance depends on shell cover and date, but the effect of date on burrow abundance depends on temperature. | (9) Shell cover + date + temperature + temperature × date |
| Burrow abundance depends on the habitat type and date. | (10) Habitat + date |
| Burrow abundance depends on habitat type and date, but the effect of date on burrow abundance depends on temperature. | (11) Habitat + date + temperature + temperature × date |
| Burrow abundance depends on whether a nest is present and date. | (12) Point type + date |
| Burrow abundance depends on whether a nest is present and date, but the effect of nest presence on burrow abundance depends on shell cover. | (13) Point type + shell cover + date + point type × shell cover |
| Burrow abundance depends on whether a nest is present and date, but the effect of date on burrow abundance depends on the temperature. | (14) Point type + date + temperature + temperature × date |
| Burrow abundance depends on whether a nest is present and date, but the effect of nest presence on burrow abundance depends on shell cover and the effect of date on burrow abundance depends on temperature. | (15) Point type + shell cover + temperature + date + point type × shell cover + temperature × date |
| Burrow abundance is explained by the environmental conditions, including the temperature, the level of shell cover, and the habitat type of the survey point. | (16) Temperature + shell cover + habitat |
| Burrow abundance is explained by the environmental conditions, plus the date. | (17) Temperature + shell cover + habitat + date |
| Burrow abundance is explained by the environmental conditions, but the effect of temperature depends on the date. | (18) Temperature + date + shell cover + habitat + temperature × date |
| Burrow abundance is explained by habitat type and whether a nest is present. | (19) Habitat + point type |
| Burrow abundance is explained by habitat type and whether a nest is present, but the effect of nest presence depends on shell cover. | (20) Habitat + point type + shell cover + point type × shell cover |
| Burrow abundance is explained by habitat type, whether a nest is present, and date. | (21) Habitat + point type + date |
| Burrow abundance is explained by habitat type, whether a nest is present, and date, but the effect of nest presence on burrow abundance depends on shell cover | (22) Habitat + point type + shell cover + date + point type × shell cover |
| Burrow abundance is explained by habitat type, whether a nest is present, and date, but the effect of date on burrow abundance depends on temperature. | (23) Habitat + point type + date + temperature + temperature × date |
| Burrow abundance is explained by habitat, whether a nest is present, shell cover, temperature, and date. | (24) Habitat + point type + shell cover + temperature + date |
| Burrow abundance is explained by habitat type, whether a nest is present, and date, but the effect of nest presence on burrow abundance depends on shell cover and the effect of date on burrow abundance depends on temperature (i.e., global model). | (25) Habitat + point type + shell cover + temperature + date + point type × shell cover + temperature × date |
| Burrow abundance is explained only by point-specific stochasticity and no other predictor variables. (i.e., Null model). | (26) 1 |
