# Supplementary material for: Biotic and abiotic factors affecting Atlantic ghost crab (Ocypode quadrata) spatiotemporal activity at an important shorebird nesting site in Virginia
Source: PLoS One. 2024 Aug 13;19(8):e0307821. doi: 10.1371/journal.pone.0307821 (PMC11321587; doi:10.1371/journal.pone.0307821)
Supplement: S2 Table — The beta estimate, standard error, and 95% confidence interval for each parameter in the top-ranked model are listed. (DOCX) [file pone.0307821.s002.docx]

| **Model parameter** | **β^a^** | **SE^b^** | **95% confidence interval** | |
| --- | --- | --- | --- | --- |
|  |  |  | **Lower limit** | **Upper limit** |
| Zero-inflated intercept | -3.38 | 0.04 | -4.17 | -2.59 |
| Zero-inflated date | -1.87 | 0.35 | -2.54 | -1.19 |
| Conditional intercept | 1.98 | 0.26 | 1.46 | 2.49 |
| Backdune flat habitat | 0.67 | 0.33 | 0.03 | 1.32 |
| Dune habitat | 0.33 | 0.28 | -0.21 | 0.88 |
| Overwash habitat | 0.16 | 0.30 | -0.43 | 0.75 |
| Beach habitat | -0.66 | 0.30 | -1.26 | -0.07 |
| Berm habitat | -0.24 | 0.35 | -0.92 | 0.44 |
| Intertidal zone habitat | -0.72 | 0.52 | -1.74 | 0.30 |
| Piping plover nest | 0.58 | 0.51 | -0.42 | 1.58 |
| American oystercatcher nest | -0.02 | 0.26 | -0.51 | 0.49 |
| Sparse shell | 0.27 | 0.19 | -0.11 | 0.65 |
| Heavy shell | -0.21 | 0.23 | -0.67 | 0.25 |
| Temperature | 0.27 | 0.04 | 0.19 | 0.35 |
| Date | 0.09 | 0.04 | 0.02 | 0.17 |
| Piping plover nest x sparse shell | -1.59 | 0.58 | -2.73 | -0.44 |
| Piping plover nest x heavy shell | -2.22 | 1.04 | -4.25 | -0.19 |
| American oystercatcher nest x sparse shell | -0.41 | 0.31 | -1.02 | 0.20 |
| American oystercatcher nest x heavy shell | -0.68 | 0.42 | -1.51 | 0.14 |
| Date x temperature | -0.06 | 0.04 | -0.13 | 0.01 |

^a^ β = beta estimate. A positive beta estimate indicates a positive effect on ghost crab burrow abundance, while a negative beta estimate indicates a negative effect on ghost crab burrow abundance.

^b^ SE = standard error
