# Supplementary material for: Biotic and abiotic factors affecting Atlantic ghost crab (Ocypode quadrata) spatiotemporal activity at an important shorebird nesting site in Virginia
Source: PLoS One. 2024 Aug 13;19(8):e0307821. doi: 10.1371/journal.pone.0307821 (PMC11321587; doi:10.1371/journal.pone.0307821)
Supplement: Attachment — Submitted filename: Response to Reviewers.docx [file pone.0307821.s003.docx]

**PONE-D-24-09865**

**Biotic and abiotic factors affecting Atlantic ghost crab ( *Ocypode quadrata* ) spatiotemporal activity at an important shorebird nesting site in Virginia**

**PLOS ONE**

**Editorial Comments and Responses**

*We thank the two reviewers and	Academic Editor for their thoughtful comments and constructive criticism which have improved this manuscript. We provide our responses to the journal requirements and all reviewer comments in italics below.*

**Journal requirements:**

**When submitting your revision, we need you to address these additional requirements.**

**1. Please ensure that your manuscript meets PLOS ONE's style requirements, including those for file naming. The PLOS ONE style templates can be found at**

**https://journals.plos.org/plosone/s/file?id=wjVg/PLOSOne_formatting_sample_main_body.pdf and**

**https://journals.plos.org/plosone/s/file?id=ba62/PLOSOne_formatting_sample_title_authors_affiliations.pdf**

*Thank you for providing these resources. We have reviewed the submission guidelines and have ensured that the manuscript meets PLOS ONE’s style requirements outlined in the provided links.*

**2. When completing the data availability statement of the submission form, you indicated that you will make your data available on acceptance. We strongly recommend all authors decide on a data sharing plan before acceptance, as the process can be lengthy and hold up publication timelines. Please note that, though access restrictions are acceptable now, your entire data will need to be made freely accessible if your manuscript is accepted for publication. This policy applies to all data except where public deposition would breach compliance with the protocol approved by your research ethics board. If you are unable to adhere to our open data policy, please kindly revise your statement to explain your reasoning and we will seek the editor's input on an exemption. Please be assured that, once you have provided your new statement, the assessment of your exemption will not hold up the peer review process.**

*Since submission of the manuscript to PLOS ONE, we have made the data publicly available via the Environmental Data Initiative (EDI) Data Portal. The data was published on 15 March 2024 and is available for download here: https://doi.org/10.6073/pasta/1f1117a917de507802f1acb4d5cf42ef*

*We have entered the full Data Availability Statement that includes the name of the database and location of the data in the PLOS ONE submission form. Additionally, we have cited the data in the methods section on* ***line 181****.*

**3. Please include your full ethics statement in the ‘Methods’ section of your manuscript file. In your statement, please include the full name of the IRB or ethics committee who approved or waived your study, as well as whether or not you obtained informed written or verbal consent. If consent was waived for your study, please include this information in your statement as well.**

*Thank you for the opportunity to improve the clarity on the ethics of our study. As requested, we have included our full ethics statement in the ‘Methods’ section of our manuscript file on* ***lines 230–245****. To summarize:*

*This project was an observational field study that included observations of some vertebrate species (i.e., shorebirds), one of which is a federally- and state-protected species (i.e., the piping plover* Charadrius melodus*). We have revised our ethics statement to include the required information for both animal research and observational and field studies, as specified in the submission guidelines for PLOS ONE. Our ethics statement includes:*

- *The full names and numbers, where applicable, as well as the full name of the issuing agency/organization, for all permits under which we conducted the research. (This was included in the ethics statement from the original submission and is unchanged).*
- *The full name of the IACUC (i.e., Institutional Animal Care and Use Committee) office that approved the research, and the associated protocol number. (This was included in the ethics statement from the original submission and is unchanged).*
- *A statement on how one species in our study, the piping plover, is a protected species. (This was added in revisions on* ***lines 235–237****).*
- *A statement on landownership for the study site, including that the island is jointly owned and managed by a private non-governmental organization (i.e., The Nature Conservancy) and a federal land management agency (i.e., U.S. Fish and Wildlife Service). (This was included in the ethics statement from the original submission and is unchanged). Additionally, we include a statement that public access to the site is seasonally restricted by the managing organization/agency so that the public cannot access land above the intertidal zone or outside designated pathways. (This was added in revisions on* ***lines 241–243****).*
- *A statement about how our research followed all applicable ethical guidelines for research involving wild avian species, as presented in the Ornithological Council’s* Guidelines to the Use of Wild Birds in Research*. (This was included in the ethics statement from the original submission; however, we included a citation for the* Guidelines *in revisions on* ***line 245*** *and provide the full citation in* ***reference 53*** *on* ***lines 565–567****).*

*We do not include an IRB (i.e., Institutional Review Board) protocol number or statement on consent (written or verbal), as this project did not include human subject research and thus did not involve human study participants.*

**4. We note that Figure 1 in your submission contain [map/satellite] images which may be copyrighted. All PLOS content is published under the Creative Commons Attribution License (CC BY 4.0), which means that the manuscript, images, and Supporting Information files will be freely available online, and any third party is permitted to access, download, copy, distribute, and use these materials in any way, even commercially, with proper attribution. For these reasons, we cannot publish previously copyrighted maps or satellite images created using proprietary data, such as Google software (Google Maps, Street View, and Earth). For more information, see our copyright guidelines: http://journals.plos.org/plosone/s/licenses-and-copyright.**

**We require you to either (1) present written permission from the copyright holder to publish these figures specifically under the CC BY 4.0 license, or (2) remove the figures from your submission:**

a. **You may seek permission from the original copyright holder of Figure 1 to publish the content specifically under the CC BY 4.0 license. We recommend that you contact the original copyright holder with the Content Permission Form (http://journals.plos.org/plosone/s/file?id=7c09/content-permission-form.pdf) and the following text: “I request permission for the open-access journal PLOS ONE to publish XXX under the Creative Commons Attribution License (CCAL) CC BY 4.0 (http://creativecommons.org/licenses/by/4.0/). Please be aware that this license allows unrestricted use and distribution, even commercially, by third parties. Please reply and provide explicit written permission to publish XXX under a CC BY license and complete the attached form.” Please upload the completed Content Permission Form or other proof of granted permissions as an "Other" file with your submission. In the figure caption of the copyrighted figure, please include the following text: “Reprinted from [ref] under a CC BY license, with permission from [name of publisher], original copyright [original copyright year].”**
b. **If you are unable to obtain permission from the original copyright holder to publish these figures under the CC BY 4.0 license or if the copyright holder’s requirements are incompatible with the CC BY 4.0 license, please either i) remove the figure or ii) supply a replacement figure that complies with the CC BY 4.0 license. Please check copyright information on all replacement figures and update the figure caption with source information. If applicable, please specify in the figure caption text when a figure is similar but not identical to the original image and is therefore for illustrative purposes only. The following resources for replacing copyrighted map figures may be helpful: USGS National Map Viewer (public domain): http://viewer.nationalmap.gov/viewer/, The Gateway to Astronaut Photography of Earth (public domain): http://eol.jsc.nasa.gov/sseop/clickmap/, Maps at the CIA (public domain): https://www.cia.gov/library/publications/the-world-factbook/index.html and https://www.cia.gov/library/publications/cia-maps-publications/index.html, NASA Earth Observatory (public domain): http://earthobservatory.nasa.gov/, Landsat: http://landsat.visibleearth.nasa.gov/, USGS EROS (Earth Resources Observatory and Science (EROS) Center) (public domain): http://eros.usgs.gov/#, Natural Earth (public domain): http://www.naturalearthdata.com/**

*Thank you for the additional information regarding PLOS ONE’s policy related to publishing copyrighted maps or satellite imagery. We checked the copyright status of all data included in Figure 1. Figure 1 includes the following data:*

- *A basemap of the United States obtained from the U.S. Census Bureau’s Topologically Integrated Geographic Encoding and Referencing (TIGER) database. These data have no access constraints, are not copyrighted, and are free to use in publication as long as acknowledgement is given to the U.S. Census Bureau.*

*The metadata for the basemap (*[*https://catalog.data.gov/dataset/tiger-line-shapefile-current-nation-u-s-state-and-equivalent-entities*](https://catalog.data.gov/dataset/tiger-line-shapefile-current-nation-u-s-state-and-equivalent-entities)*) explicitly states: “Use Constraints: The TIGER/Line Shapefile products are not copyrighted however TIGER/Line and Census TIGER are registered trademarks of the U.S. Census Bureau. These products are free to use in a product or publication, however acknowledgement must be given to the U.S. Census Bureau as the source. The boundary information in the TIGER/Line Shapefiles are for statistical data collection and tabulation purposes only; their depiction and designation for statistical purposes does not constitute a determination of jurisdictional authority or rights of ownership or entitlement and they are not legal land descriptions. Coordinates in the TIGER/Line shapefiles have six implied decimal places, but the positional accuracy of these coordinates is not as great as the six decimal places suggest.”*

*As per the constraints, we provide acknowledgement to the U.S. Census Bureau in the caption for Figure 1 on* ***line 102.***

- *A basemap of Virginia obtained from the Virginia Geographic Information Network (VGIN) Virginia GIS Clearinghouse via the Virginia Administrative Boundaries download (*[*https://vgin.vdem.virginia.gov/datasets/777890ecdb634d18a02eec604db522c6/about*](https://vgin.vdem.virginia.gov/datasets/777890ecdb634d18a02eec604db522c6/about)*). We have confirmed with a representative from VGIN that all data distributed through the Virginia GIS Clearinghouse can be used and redistributed without restriction or license. We have included a screenshot of that correspondence here, but also upload proof of correspondence as an ‘Other’ file with our submission, as requested.*

*Because we acknowledged the U.S. Census Bureau in our Figure 1 caption, we have also acknowledged VGIN in the figure caption, on* ***line 103.***

**Reviewers' comments:

Reviewer's Responses to Questions**

**Comments to the Author

1. Is the manuscript technically sound, and do the data support the conclusions?

The manuscript must describe a technically sound piece of scientific research with data that supports the conclusions. Experiments must have been conducted rigorously, with appropriate controls, replication, and sample sizes. The conclusions must be drawn appropriately based on the data presented.**

**Reviewer #1: Partly**

**Reviewer #2: Yes**

**2. Has the statistical analysis been performed appropriately and rigorously?**

**Reviewer #1: No**

**Reviewer #2: No**

**3. Have the authors made all data underlying the findings in their manuscript fully available?

The PLOS Data policy requires authors to make all data underlying the findings described in their manuscript fully available without restriction, with rare exception (please refer to the Data Availability Statement in the manuscript PDF file). The data should be provided as part of the manuscript or its supporting information, or deposited to a public repository. For example, in addition to summary statistics, the data points behind means, medians and variance measures should be available. If there are restrictions on publicly sharing data—e.g. participant privacy or use of data from a third party—those must be specified.**

**Reviewer #1: Yes**

**Reviewer #2: No**

**4. Is the manuscript presented in an intelligible fashion and written in standard English?

PLOS ONE does not copyedit accepted manuscripts, so the language in submitted articles must be clear, correct, and unambiguous. Any typographical or grammatical errors should be corrected at revision, so please note any specific errors here.**

**Reviewer #1: Yes**

**Reviewer #2: Yes**

**5. Review Comments to the Author

Please use the space provided to explain your answers to the questions above. You may also include additional comments for the author, including concerns about dual publication, research ethics, or publication ethics. (Please upload your review as an attachment if it exceeds 20,000 characters)**

**Reviewer #1:**

**"I would appreciate a revision of the map in Fig. 1, which could display less of the USA but should provide much higher detail of Metompkin Island. Indeed, readers of the paper may be interested in better visualizing the study area. Therefore, I suggest zooming in much more on that island. It's not even necessary to show the entire island, but the position of the surveyed area should be identifiable on the map. If this proves to be practically impossible, at least the coordinates of the limits of the 3 km-long stretch of the island used for the survey should be added to ensure the research can be fully replicated in the future by other researchers.**

*Thank you for this comment, as we think your suggestions greatly improve this figure. We’ve revised Figure 1 according to the suggested revisions provided here. We have revised the inset map to show more detail of Metompkin Island and less detail of the United States. Additionally, we have indicated the location of the study area on the inset map.*

*Additionally, as suggested, we’ve provided the coordinates for the extent of the study area in-text in the study area description within the manuscript on* ***lines 121–122****. We also chose to provide coordinates for the centroid of the study area.*

**In the Abstract, it is stated, "We observed burrows at all nest sites in our study area but found fewer burrows at nest sites than random sites." This was an unexpected result for the authors. Consequently, they suggested that "Ghost crabs may avoid shorebird nest sites due to aggressive defensive behaviors from incubating adults or differences in microhabitat characteristics selected by shorebirds versus ghost crabs." This point is intriguing for readers because the study was not solely motivated by interest in ghost crab behavior and ecology but primarily due to the threat posed by the crabs to the reproductive success of endangered birds. Therefore, it's expected that the available data would be utilized, if possible, to test which of the two suggested causes was involved. While the behavior of the nesting birds wasn't studied, as far as I'm aware, the microhabitat was examined. Microhabitat is significant because other studies have demonstrated that piping plovers prefer to nest in areas with above-average shell cover, while crabs prefer to dig in areas with a low amount of shells, where digging is easier for them. However, the interaction between the presence of shorebird nests and the amount of shell cover wasn't tested. I speculate that this issue could be investigated by incorporating, in the glmm model, a dependent variable: the interaction term between substrate type and the presence of bird nests. If there was a reason not to test this interaction, authors should explain why. As a reader, I'd also like to understand the extent to which the increase in the number of burrows depends on the likely interaction of date and temperature. Therefore, I suggest adding this interaction term to the glmm model as well. I understand that the authors obviously didn't include all possible interaction terms between predictor variables and that they chose to test only a set of 17 models based on a priori hypotheses about the effect of each predictor variable, but the suggested two interactions seem ecologically sound.**

*Thank you! We appreciate the time and thought that the reviewer put into formulating this suggestion, because we believe that including the two suggested interaction terms has the potential to improve the analysis and test our assumption of the mechanism behind our findings.*

*The reviewer is correct that we did not quantify aggressive behavior of the nesting birds towards ghost crabs, as this study was not set up to allow for an observational study of nesting bird behavior. However, as the reviewer mentioned, we have the data to further investigate our hypothesis on differences between microhabitat characteristics used by ghost crabs vs nesting shorebirds.*

*We revised our analysis based on the suggestions provided. First, we included an interaction term for point type and shell cover and an interaction term for date and temperature in our global model. We then revised our model set to now include 26 models, to account for adding two interaction terms to the global model. We have included a list of those models in* ***S1 Table.*** *We used the same model selection procedures as described in the prior manuscript draft to rank the models and select a top model. The global model (this time, including the interaction terms) came out as the top-ranked model. We considered one additional model (i.e., the model with all variables as additive effects only, and no interactive effects) to be competitive, but because the interaction effects of multiple variables are only in one of the top models, we did not choose to model average and only report parameter estimates from the top-ranked model.*

*We’ve also revised the ‘Results’ section to reflect changes to our results after modifying the analysis. To summarize:*

- *Our top-ranked model was the global model, which included the random effect, both interaction effects, and five additive effects (****lines 253–255****).*
- *The interaction between date and temperature was inconclusive, as the 95% confidence intervals overlapped zero (****lines 276–277****). We found that there were greater absences of crab burrows at early dates in the study (****lines 275–276****), so it is possible there’s a finer-scale association between temperature, date, and crab burrow abundance than what we were able to measure in this study. Thus, we highlight this as an area for future study* ***(lines 378–381****).*
- *The interaction between point type and shell cover was important for piping plovers, but not American oystercatchers. Specifically, burrow counts were lower at piping plover nests with sparse to heavy shell cover, relative to random points with no shell cover (****lines 278–281****).*
- *Backdune flats had more burrows relative to the backbarrier habitat and beaches had fewer burrows relative to the backbarrier habitat (****lines 293–294****).*

**Additionally, as a reader, I'm curious to know whether the diameter of the burrows was measured, in addition to the number of burrows. I presume that the size of crabs would be significant in their interaction with birds. Large ghost crabs pose more danger to eggs and chicks, while tiny crabs with a carapace length of 1 cm should not be dangerous or could even be considered as food for birds. Therefore, was the size of the burrows measured? If not, I expect the authors to add a brief note in the Methods section explaining why it wasn't done despite its potential significance.**

*Thank you for noting this important detail that we failed to include in our ‘Methods’ section. As mentioned in the comment, we were particularly interested in quantifying the activity of crabs that were large enough to predate shorebird nests or chicks. We only counted burrows 2.5 cm or larger in diameter in our study. We often used only a visual estimate to determine size, as we wanted to restrict the amount of time that we spent around nests to limit disturbance to incubating adults. For burrows that weren’t obviously over- or undersized, we did measure the entrance of the burrow. We’ve included these details in the manuscript under ‘Field methods,’ now on* ***lines 157–159****.*

**Not much information is provided on the glmm used for the analysis. For example, which response distribution and error distribution were used? Please add all relevant information that can be useful for the reader.**

*Thank you for this comment, we agree that we needed to provide further information on the model fit in our analysis. To summarize, we fit glmms to predict ghost crab burrow abundance (our response variable) in relation to date, temperature, habitat type, shell cover, and point type (****lines 183–194****). Our models also included a random effect of point ID to account for repeated measures (****line 208–209****). Because ghost crab burrow abundance is measured on a continuous scale [0, ∞) and because we detected evidence of overdispersion, we fit the glmms in the original submission with a negative binomial error distribution with a log link function (the default link function).*

*However, when we added the interactive effects to our model and checked assumptions, we found that the more complex model violated assumptions about zero-inflation. As a result, we fit our revised models as zero-inflated negative binomial models in which a zero-inflated component of the model predicts the probability of observing excess zeros, while a conditional component of the model predicts burrow counts conditional on the zero inflated component (****lines 202–210****). We fit glmms using the R package glmmTMB (****line 200*** *in ‘Methods’;* ***reference 39****, located on* ***line 529–532****). Our global model was encoded as:*

*glmmTMB(burrow.count ~ (1| point.id) + habitat + point.type+ shell.cover + date + temp +*

*point.type* × *shell.cover + date* × *temp,*

*ziformula = ~ date,*

*family = nbinom2, data = count)*

*Where “burrow.count ~ (1| point.id) + habitat + point.type+ shell.cover + date + temp + point.type × shell.cover + date × temp” was the conditional model component, and “ziformula = ~ date” was the zero-inflated model component (as per* ***ref 39****). Fixed effects within the conditional model component changed depending on the candidate model, as outlined in* ***Table S1****.*

**As I'm not an expert in statistics, I would appreciate it if the authors could provide a key in the Methods section on how to evaluate when an effect size is considered significant. For example, how significant are the apparently very low beta values of 0.05 and 0.08 for temperature and date (line 189)?**

*Thank you for this feedback to ensure our methods are approachable for readers with a range of familiarity with our methods. We provide a general overview of our statistical framework below, with additional references to support our analytical decisions. Following this explanation, we detail the revisions in our manuscript intended to improve the clarity behind our analytical approaches.*

*In our manuscript, we use an information-theoretic approach to explore trends in the data. Similar to frequentist (p-value) approaches, we construct possible models representing biological hypotheses. However, information theory uses AIC (i.e., Akaike’s information criterion) to rank competing models whereas a frequentist approach uses p-values to identify significant variables in a model (Burnham and Anderson 2004). More specifically, we use second-order AIC (i.e., AIC_c_), which is preferred for smaller sample sizes (typically n < 40 x number of parameters; AIC_c_ will approximate AIC at large sample sizes) and is interpreted the same as AIC (Hurvich and Tsai 1991). Since we are using an information-theoretic approach, we cannot determine variable significance, as significance testing with p-values is incompatible with an information-theoretic framework (Burnham and Anderson 2004). Instead, we use AIC_c_ to rank models and we identify the model that best describes the data. While there are several ways authors may select the ‘best’ model from a list of plausible, candidate models, we identify the ‘best’ model as that with the greatest model weight (i.e., the relative likelihood the model fits the data and is bound by 0 [poor model fit of the data] and 1 [perfect model fit of the data]). When the weight of the top-ranked model was < 0.9, we then considered models that were within 2 AIC_c_ units (i.e., ΔAIC_c_ ≤ 2.0) to be equally plausible options to the top-ranked model (Burnham and Anderson 2004).*

*After selecting the model through AIC_c_, we then examined the parameters within the model to determine effects. Positive beta coefficient indicates a positive relationship between the predictor and response variable and a negative beta coefficient indicates a negative relationship between a predictor and response variable (Zar 2010). A larger magnitude indicates a stronger relationship, and a smaller magnitude indicates a weaker relationship (Zar 2010). The confidence intervals, which we use for statistical inference, show the range of values that contain the true mean (95% probability; Cumming et al. 2007). When confidence intervals of a beta coefficient are bound between two positive (e.g., 2 – 4) or two negative values (e.g., -2 – -4), we would interpret that the parameter has a statistically meaningful positive or negative effect on the response variable. However, when the confidence interval of a beta coefficient includes (i.e., overlaps with) zero (e.g., 0 – 2, -2 – 2), then there is uncertainty about the effect of the predictor variable on the response variable, as there is a chance that the true effect of the predictor variable on the response variable could be zero (i.e., no meaningful effect; Cumming et al. 2007).*

*We appreciate the opportunity to clarify our analytical methods! We see how the language in our first submission (e.g., “testing” variables) can imply statistical testing via p-values. To better align with our analytical framework we describe above, we’ve revised both the ‘Methods’ section and caption for Figure 3 to provide a better explanation of how to interpret our results. To summarize:*

- *We clarified that we used an information-theoretic approach to model selection to compare 26 candidate models and select a model for inference on* ***lines 215–216****.*
- *We clarified that we used second-order Akaike’s information criterion corrected for small sample size to rank the models on* ***lines 220–221****.*
- *We explained how we interpreted beta coefficients for parameters in our model on* ***lines 224–227****.*
- *We explained how we identified meaningful variables using confidence intervals on* ***lines 227–229****.*
- *We added citations for all references provided in our explanation above, as needed (see* ***references 48, 49, 50 and 52****).*

*References in this response (in order of mention):*

*Burnham KP, Anderson DR. Multimodel inference: Understanding AIC and BIC in model selection. Socio Methods Res. 2004;33: 261–304. doi:10.1177/0049124104268644*

*Hurvich CM, Tsai C-L. Bias of the corrected AIC criterion for underfitted regression and time series models. Biometrika. 1991;78: 499–509. doi:10.1093/biomet/78.3.499*

*Zar JH. Biostatistical Analysis, 5th ed. Upper Saddle River (NJ): Pearson; 2010*

*Cumming G, Fidler F, Vaux DL. Error bars in experimental biology. J Cell Biol. 2007;177: 7–11. doi:10.1083/jcb.200611141*

**Again, I'm not an expert in statistics, but I wonder why the beta effect sizes of dune and back dune flat habitat reported in the Results (0.05+-0.01 and 0.05+-0.01) (line 191) do not correspond to what can be seen in Fig. 2. Please correct or explain.**

*Thank you for the opportunity to clarify. In our first submission, the beta and SE for dune and back dune habitats were x (mean) ± x (SE), as the reviewer notes. In our original Fig. 2 (now* ***Fig. 3*** *due to the addition of a new figure), we present the mean beta with the 95% confidence intervals. From Cumming et al. (2007) (****reference 52*** *in the manuscript), we calculate confidence intervals as:*

$$\mu\pm t_{(n-1)}\cdot SE$$

*where we use the beta value as the mean (*$\mu$*), and SE is the standard error.* $t_{(n-1)}$ *is the critical value. Because we are interested in a 95% confidence interval, we use the corresponding critical t-value for 95%, which is equal to 1.96 (Zar 2010;* ***reference 50*** *in the manuscript).*

*We highlight that we have revised this figure (now* ***Fig. 3****, due to the addition of a new figure) and the associated text in ‘Results’ based on new model results. We note that the math remains the same, and thus applying the formula above yields the following values:*

| ***Parameter*** | ***β*** | ***SE*** | ***95% confidence interval*** | | ***95% CI*** |
| --- | --- | --- | --- | --- | --- |
|  |  |  | ***Lower limit*** | ***Upper limit*** | ***includes zero?*** |
| *Intercept* | *1.98* | *0.26* | *1.46* | *2.49* | *No* |
| *Beach habitat* | *-0.66* | *0.30* | *-1.26* | *-0.07* | *No* |
| *Berm habitat* | *-0.24* | *0.35* | *-0.92* | *0.44* | *Yes* |
| *Dune habitat* | *0.33* | *0.28* | *-0.21* | *0.88* | *Yes* |
| *Backdune flat habitat* | *0.67* | *0.33* | *0.03* | *1.32* | *No* |
| *Intertidal zone habitat* | *-0.72* | *0.52* | *-1.74* | *0.30* | *Yes* |
| *Overwash habitat* | *0.16* | *0.30* | *-0.43* | *0.75* | *Yes* |
| *American oystercatcher nest* | *-0.02* | *0.26* | *-0.51* | *0.49* | *Yes* |
| *Piping plover nest* | *0.58* | *0.51* | *-0.42* | *1.58* | *Yes* |
| *Sparse shell* | *0.27* | *0.19* | *-0.11* | *0.65* | *Yes* |
| *Heavy shell* | *-0.21* | *0.23* | *-0.67* | *0.25* | *Yes* |
| *Date* | *0.09* | *0.04* | *0.02* | *0.17* | *No* |
| *Temperature* | *0.27* | *0.04* | *0.19* | *0.35* | *No* |
| *American oystercatcher nest x sparse shell* | *-0.41* | *0.31* | *-1.02* | *0.20* | *Yes* |
| *Piping plover nest x sparse shell* | *-1.59* | *0.58* | *-2.73* | *-0.44* | *No* |
| *American oystercatcher nest x heavy shell* | *-0.68* | *0.42* | *-1.51* | *0.14* | *Yes* |
| *Piping plover nest x heavy shell* | *-2.22* | *1.04* | *-4.25* | *-0.19* | *No* |
| *Date x temperature* | *-0.06* | *0.04* | *-0.13* | *0.01* | *Yes* |
| *Zero-inflated intercept* | *-3.38* | *0.04* | *-4.17* | *-2.59* | *No* |
| *Zero-inflated date* | *-1.87* | *0.35* | *-2.54* | *-1.19* | *No* |

*To aid in transparency behind calculating 95% confidence intervals, we have added reference to Cumming et al. (2007) in* ***line 227*** *in the ‘Methods,’ and* ***lines 563–564*** *in ‘References.’*

*Additionally, we note that a modified version of the table above is now included as S2 Table, as per PLOS ONE’s requirements to include the full results of a regression analysis as supporting information.*

**Is the SE of the effect size of piping plover nest really 0.01 as stated in the results (line 214)? It appears to be different in Fig. 2. Please verify.**

*Thank you for this comment. We interpret this comment similar to the previous comment made above. Above, we provide greater explanation as to how we calculate confidence intervals from the betas and their corresponding standard errors. The same formula was applied to our original results and Figure 2.*

*We’ve revised this figure (now* ***Fig. 3****, due to the addition of a new figure) and the associated text in ‘Results’ based on new model results. We note that the SEs and their corresponding confidence intervals can be double-checked above in the table using the formula from Cumming et al. (2007) provided above.*

**Regarding the Acknowledgments, I find it peculiar that "In addition to the authors listed for this study, we thank staff at the University of Virginia Coastal Research Station." Does this mean that the authors are thanking themselves? Later, D. Fraser is thanked. Is he the same D. Fraser who is one of the co-authors?"**

*We appreciate this careful review of the Acknowledgements section. We understand how the wording of the first sentence of the Acknowledgements is confusing, so we have modified the sentence to read as: “We thank staff at the University of Virginia Coastal Research Station, as well as S. Ritter and D. Fraser from the Virginia Tech Department of Fish and Wildlife Conservation, for their logistical and intellectual support over the course of the study.” Additionally, we would like to note that none of the authors are affiliated with the University of Virginia Coastal Research Station. Virginia Tech (the institution of seven coauthors) is a different institution than the University of Virginia, which owns and administers the Coastal Research Station.*

*We thanked D. Fraser in the Acknowledgements section for his logistical and intellectual support throughout the study. We can confirm that D. Fraser, as thanked in the Acknowledgements section, is not the same person as the coauthor, James D. Fraser.*

**Reviewer #2:**

**Overall impression:

This study on Atlantic ghost crabs and their potential impact on beach-nesting shorebirds is well-written and addresses an important, yet under-appreciated topic of conservation interest. I have some concerns regarding the analytical methods, however, which could be addressed with the addition of significant clarification and justification in the manuscript. Specifically, while the abstract outlines the methodology and key findings regarding ghost crab activity and habitat selection, a more thorough account of hypothesis testing and statistical significance would enhance the manuscript's rigor. Overall, the study contributes valuable insights into ghost crab ecology and its implications for shorebird conservation, but further refinement of the analysis and interpretation is needed to strengthen the manuscript.**

*Thank you for your comments on this manuscript. We appreciate that you found our manuscript to address an important conservation topic. We agree that additional clarification and justification is needed for the analytical methods—we provide more detailed responses to the suggested revisions below.*  **Major comments:

1) The authors should provide further clarification on the methods used for counting burrows between the random point locations and the nest sites. If random point surveys were indeed a point, then a 2m radius around the outside of a nest would yield a greater surface area (based on basic geometric principles) for examination compared to a 2m radius around a point, correct? If nest size varies greatly, this would also cause variability in surface area for surveys. Perhaps the diameter of the nests are small enough that the difference in area would be negligible, but the authors should address this possibility and provide further clarification on the characteristics of shorebird nest size.**

*We appreciate the reviewer raising this question about our survey methods and providing us with the opportunity for further clarification of shorebird reproductive ecology and the methods. We’ve provided a further explanation of the methods here, but also include the same information in our manuscript revisions on* ***lines 150–157*** *.*

*We conducted surveys of ghost crab burrows within a 2 m radius of random points and shorebird (i.e., piping plover and American oystercatcher) nests in our study, meaning we counted all ghost crab burrows above a 2.5 cm size threshold (see below) within 2 m of the random point or nest point. We assumed that all nest points were located in the center of the clutch in each nest. Shorebird nests (including both piping plovers and American oystercatchers) consist of a clutch of eggs (typically 4 eggs for piping plovers and 3 eggs for American oystercatchers), where eggs are directly deposited in shallow indentations in sand.* *Shorebird nests are not large in size, no more than 10 cm in diameter for piping plovers (Elliott-Smith and Haig 2020) and 20 cm in diameter for American oystercatchers (American Oystercatcher Working Group et al. 2020). Additionally, as the nest is only a shallow scrape in sand, any ghost crab burrows located within the nest, including under the eggs, would be visible and available for counting. Thus, we do not have reason to believe that there was any variability in the surface area surveyed or visibility of crab burrows during surveys at nests versus random points.*

*We have added this information described above to the ‘Field methods’ section on* ***lines 150–157****, also pasted here: “We counted crab burrows within a 2 m radius of each nest or random sampling point (Fig 2d) during each visit. The center of the sampling point for each nest was located in the center of the clutch. Both piping plover and American oystercatcher nests consist of clutches of eggs laid in a shallow scrape in the sand, and thus the area surveyed around each nest was the same as the area surveyed around each random sampling location. Beach-nesting shorebird nests are not large in size, approximately 10 cm in diameter for piping plovers [32] and 20 cm in diameter for American oystercatchers [33]. Additionally, nests did not affect our ability to detect ghost crab burrows, as we were able to count burrows even if they were located within the shallow nest scrape.”*

*References in this response (in order of mention):*

*Elliott-Smith E, Haig SM. Piping Plover (Charadrius melodus), v. 1.0. In: Poole AF, editor. Birds of the world. Ithaca (NY): Cornell Lab of Ornithology; 2020. doi: 10.2173/bow.pipplo.01*

*American Oystercatcher Working Group, Nol E, Humphrey RC. American Oystercatcher (Haematopus palliatus), v. 1.0. In: Poole AF, editor. Birds of the world. Ithaca (NY): Cornell Lab of Ornithology; 2020. doi: 10.2173/bow.ameoys.01*

**2) The abstract and introduction state that the authors tested whether ghost crab activity was greater at shorebird nest sites than random sites, however the results presented in the manuscript do not adequately answer this question and lack testing with any level of statistical significance. While the model procedure used for analysis seems appropriate, the text is lacking proper explanation of full analysis procedures. Additionally, it does not appear that the authors have conducted any significance tests (e.g. Wald tests or likelihood ratio tests) along with their models.**

*Thank you for this comment, as it made us realize that we were not precise with our language concerning our analyses and results. We have made sure to further clarify our full analytical methods in ‘Statistical analyses’ section of the manuscript as this comment was similar to that of another reviewer. Additionally, we have changed our language in the abstract and introduction to reflect the type of analysis that we performed—please note that all instances where we used the word ‘test’ in the abstract now use the word ‘investigate’ (such as on* ***line 36****).*

*Most importantly, we have clarified that we used an information-theoretic approach to model selection, as outlined by Burnham and Anderson 2004 and discussed in Blankenship et al. 2002. Thus, we constructed a set of candidate models that included models from biologically plausible* a priori *hypotheses, a global model that included all variables of interest, and a null (i.e., intercept-only) model and ranked those models using a model selection criterion (Burnham and Anderson 2004). We specifically used second-order AIC (i.e., AIC*_c_*) which is corrected for small sample size, to rank the model (Hurvich and Tsai 1991).*

*We then identified a ‘best’ model from the list of candidate models. We note that there are several approaches to selecting a ‘best’ model, but we chose to use the ΔAIC_c_ value. We considered models that were within 2 AIC_c_ units (i.e., ΔAIC_c_ ≤ 2.0) to be equally plausible options to the top-ranked model (Burnham and Anderson 2004).*

*Finally, we used the selected ‘best’ model for inference, i.e., identifying variables that are important predictors of the response variable: ghost crab burrow counts. We examined the parameters within the model to determine the effects of each predictor variable on the response variable. A positive beta coefficient indicates a positive relationship between the predictor and response variable and a negative beta coefficient indicates a negative relationship between a predictor and response variable (Zar 2010). A larger magnitude indicates a stronger relationship, and a smaller magnitude indicates a weaker relationship (Zar 2010). We used the 95% confidence interval of each parameter for statistical inference, i.e., when the confidence interval was bound between two positive or negative values, we interpreted the parameter as having a meaningful positive or negative effect on the response variable (Cummings et al. 2007).*

*We note that because we used this approach over a hypothesis testing approach, we did not in fact* ***test*** *whether ghost crab activity was greater at shorebird nesting sites than random sites. Additionally, we did not* ***test*** *the effect of any other variable. We recognize that the use of the word ‘test’ implies significance testing, when in fact that was not our goal. Instead, we aimed to* ***investigate*** *the potential effect of different predictor variables on a response variable (i.e., ghost crab burrow counts).*

*We chose the information-theoretic approach over a hypothesis testing approach (i.e., using significance tests such as likelihood ratio tests) because it allows for us to rank multiple models based on fit and model complexity (i.e., number of parameters) and compare relative likelihoods. Johnson and Omland 2004 provide a good explanation of hypothesis testing versus model selection criteria, and we referenced it when deciding on our analytical methods.*

*We conducted a Wald test on our top model (please note: our top model has changed, since we have included two interaction effects in the global model following the suggestions of another reviewer). The p-value for our interactive effect between point type and shell (as suggested by another reviewer) is 0.059. However, we are choosing not to report this in our manuscript as we are making an effort not to mix the information-theoretic and hypothesis testing approaches, as per standardized practices outlined by Burnham and Anderson 2004. Since we are using an information-theoretic approach, we cannot determine variable significance, as significance testing with p-values is incompatible with an information-theoretic framework (Burnham and Anderson 2004). Instead, we identify predictor variables as having an important effect on ghost crab burrow counts based on if the 95% confidence interval for their beta coefficient overlaps with zero.*

*In our revisions, we have clarified our methods based on the explanation that we have provided here. We have stated that we used an information-theoretic approach to model investigation and provided a step-by-step explanation of how we selected our top model and interpreted which variables were important predictors of the response variable. We have also made sure to cite in the manuscript all sources referenced in our response; specifically, we added citations for Blankenship et al. 2002, Hurvich and Tsai 1991, Johnson and Omland 2004. Additionally, we added citations for Zar 2010 and Cumming et al. 2007. To summarize the changes that we have made:*

- *We clarified that we used an information-theoretic approach to model selection to compare 26 candidate models and select a model for inference on* ***lines 215–216****.*
- *We clarified that we used second-order Akaike’s information criterion corrected for small sample size to rank the models on* ***lines 220–221****.*
- *We explained how we interpreted beta coefficients for parameters in our model on* ***lines 224–227****.*
- *We explained how we identified meaningful variables using confidence intervals on* ***lines 227–229****.*
- *We added citations for all references provided in our explanation above, as needed (see* ***references 44, 46, 48, 49, 50 and 52****).*

*References in this response (in order of mention):*

*Burnham KP, Anderson DR. Multimodel inference: Understanding AIC and BIC in model selection. Socio Methods Res. 2004;33: 261–304. doi:10.1177/0049124104268644*

*Blankenship EE, Perkinsb MW, Johnsonc RJ. The information-theoretic approach to model selection: description and case study. Conference on Applied Statistics in Agriculture. 2002. doi:10.4148/2475-7772.1200*

*Hurvich CM, Tsai C-L. Bias of the corrected AIC criterion for underfitted regression and time series models. Biometrika. 1991;78: 499–509. doi:10.1093/biomet/78.3.499*

*Zar JH. Biostatistical Analysis, 5th ed. Upper Saddle River (NJ): Pearson; 2010*

*Cumming G, Fidler F, Vaux DL. Error bars in experimental biology. J Cell Biol. 2007;177: 7–11. doi:10.1083/jcb.200611141*

*Johnson JB, Omland KS. Model selection in ecology and evolution. Trends Ecol Evol. 2004;19: 101–108. doi:10.1016/j.tree.2003.10.013*

**Upon inspection of Figure 2, it appears that many of the model effects are not statistically significant (i.e. the confidence intervals include zero). If this is not the proper interpretation, the authors should take care to provide further details and explanation in the methods and results sections.**

*Thank you for this opportunity to clarify analytical methods. We have revised some of our candidate models based on recommendations made by another reviewer. The content originally appearing in Fig 2 (beta coefficients and their 95% confidence intervals) now appear in* ***Fig. 3*** *as we added a new figure to clarify our field sampling methods of nests and burrow counts (see new* ***Fig. 2****).*

*The following table includes updated beta coefficients, SE, and 95% confidence interval limits for our revised analysis. Additionally, we provide a column that indicates if the 95% confidence interval for a parameter includes 0. If yes, we do not consider the parameter informative. If no, we consider the parameter informative. We have included a modified version of this table as S2 Table, as per PLOS ONE’s guidelines to present full results from regression analysis.*

| ***Parameter*** | ***β*** | ***SE*** | ***95% confidence interval*** | | ***95% CI*** |
| --- | --- | --- | --- | --- | --- |
|  |  |  | ***Lower limit*** | ***Upper limit*** | ***includes zero?*** |
| *Intercept* | *1.98* | *0.26* | *1.46* | *2.49* | *No* |
| *Beach habitat* | *-0.66* | *0.30* | *-1.26* | *-0.07* | *No* |
| *Berm habitat* | *-0.24* | *0.35* | *-0.92* | *0.44* | *Yes* |
| *Dune habitat* | *0.33* | *0.28* | *-0.21* | *0.88* | *Yes* |
| *Backdune flat habitat* | *0.67* | *0.33* | *0.03* | *1.32* | *No* |
| *Intertidal zone habitat* | *-0.72* | *0.52* | *-1.74* | *0.30* | *Yes* |
| *Overwash habitat* | *0.16* | *0.30* | *-0.43* | *0.75* | *Yes* |
| *American oystercatcher nest* | *-0.02* | *0.26* | *-0.51* | *0.49* | *Yes* |
| *Piping plover nest* | *0.58* | *0.51* | *-0.42* | *1.58* | *Yes* |
| *Sparse shell* | *0.27* | *0.19* | *-0.11* | *0.65* | *Yes* |
| *Heavy shell* | *-0.21* | *0.23* | *-0.67* | *0.25* | *Yes* |
| *Date* | *0.09* | *0.04* | *0.02* | *0.17* | *No* |
| *Temperature* | *0.27* | *0.04* | *0.19* | *0.35* | *No* |
| *American oystercatcher nest x sparse shell* | *-0.41* | *0.31* | *-1.02* | *0.20* | *Yes* |
| *Piping plover nest x sparse shell* | *-1.59* | *0.58* | *-2.73* | *-0.44* | *No* |
| *American oystercatcher nest x heavy shell* | *-0.68* | *0.42* | *-1.51* | *0.14* | *Yes* |
| *Piping plover nest x heavy shell* | *-2.22* | *1.04* | *-4.25* | *-0.19* | *No* |
| *Date x temperature* | *-0.06* | *0.04* | *-0.13* | *0.01* | *Yes* |
| *Zero-inflated intercept* | *-3.38* | *0.04* | *-4.17* | *-2.59* | *No* |
| *Zero-inflated date* | *-1.87* | *0.35* | *-2.54* | *-1.19* | *No* |

*We have verified the content in our Results and Discussions sections reflect the correct interpretation of parameters. Thank you again for helping us ensure correct interpretation.*

**Minor comments:

Line 23— The first line of the abstract makes it sound like ghost crabs prey on hatched chicks and adult shorebirds, but it is clear in the other text that they are potential predators only of nests and eggs. Please clarify here to be in agreement with other sections of the text.**

*Thank you for bringing this to our attention, and we have checked the text to make sure that* ***line 26*** *is in agreement with the information that we provide later in the introduction.* ***Line 26*** *now does not mention adults, as we did not provide evidence of direct predation of shorebird adults by ghost crabs in the introduction (instead, one source documented ghost crabs injuring adults).*

***Line 26*** *now says: “Atlantic ghost crabs (*Ocypode quadrata*) are predators of beach-nesting shorebird nests and chicks on the United States’ Atlantic and Gulf coasts.”* **Line 52— causes used twice, please replace one instance**

*Thank you. We have replaced “causing” with “creating”. The part of the sentence on* ***line 54*** *now says: “… by creating a disturbance that can attract other predators…”* **Line 60— reword to “are not often targeted.” Are the authors aware of any instances where ghost crabs are targeted, aside from the one report citation from FL?**

*Thank you for this suggestion. We’ve made the suggested revision to the wording. While there may be other efforts that have targeted ghost crabs for removal as a way of improving shorebird reproductive success, we are not aware of any other published reports or studies, so we modified the sentence to reflect that.*

*These revisions are now on lines 66–69, which say: “While there is some evidence that the lethal removal of ghost crabs can improve shorebird reproductive success within the Western Atlantic Flyway (i.e., [15]), ghost crab management generally does not surface as a leading conservation strategy and warrants more attention from the conservation community.”* **Line 62—change to “evidence” from isolated studies**

*Thank you. We’ve made the suggested revision, which is now in* ***line 66*** *(“While there is some evidence…”).* **Line 70— are conditions “inhospitable” or simply outside the preferred or optimal temperature range? Consider word use here, perhaps “suboptimal” would be a better fit?**

*We appreciate the reminder of precision with language. In this sentence, we intended to discuss how ghost crabs may now be occupying latitudes where temperatures were previously either suboptimal or exceeded ghost crabs’ lethal thermal limit. For clarity, we have revised this sentence to read as “ghost crabs appear to be expanding into previously unoccupied northern latitudes.” (Now on* ***line 74****)*

**Line 72— what kind of “change” in population is being referred to here. Please be more explicit.**

*Thank you for the opportunity to clarify. We have clarified that we were referring to changes in ghost crab population abundance (****lines 75–77****), which may either increase or decrease in response to the listed activities.*  **Line 80— change “spatiotemporal” to spatial and temporal**

*Thank you. We have made the suggested revision (now on* ***line 84****: “We identified factors affecting the spatial and temporal patterns…”).*  **Methods
Line 107— Please, remove one instance of “species” to avoid repetition**

*Thank you. We have modified the sentence to remove one instance of the word “species.” The revised sentence is now on* ***lines 107–109*** *and says: “Both piping plovers and American oystercatchers are species of conservation concern, with the Atlantic coast population of the piping plover federally listed as threatened in the United States [11,26].”* **Line 130— Do the shorebird nests vary in size/diameter? This would affect the total area of surveys when using a 2m radius around a nest. Similarly for the point surveys. Please see major comment #1 above.**

*There is a small difference in nest size for piping plovers versus American oystercatchers (10 cm versus 20 cm, respectively). However, the difference in nest size does not affect the area surveys, which was a 2 m radius around the point. We counted all ghost crab burrows within that 2 m radius, including burrows that may have occurred within the nest cup (i.e., the shallow scrape). We have revised the manuscript to clarify that the nests of both species did not affect the size of the area surveyed or our ability to detect crab burrows, and we have also added a new* ***Fig 2*** *that shows the nests and a graphical representation of how we surveyed burrows within a fixed radius of each nest. We also clarified these methods and the sizes of the nests in our revisions.*

*Those revisions are located on* ***lines 150–157****, which says: “We counted crab burrows within a 2 m radius of each nest or random sampling point (Fig 2d) during each visit. The center of the sampling point for each nest was located in the center of the clutch. Both piping plover and American oystercatcher nests consist of clutches of eggs laid in a shallow scrape in the sand, and thus the area surveyed around each nest was the same as the area surveyed around each random sampling location. Beach-nesting shorebird nests are not large in size, approximately 10 cm in diameter for piping plovers [32] and 20 cm in diameter for American oystercatchers [33]. Additionally, nests did not affect our ability to detect ghost crab burrows, as we were able to count burrows even if they were located within the shallow nest scrape.”* **Statistical analysis
Line 166— What type of mixed effects model? In this case, “global” is used improperly and the type of model should be explicitly stated. I assume you are referring to a generalized linear mixed-effects model (GLMM)? The distribution and function family used in the GLMM should also be clearly described. Please, define your global model with all covariates in a separate sentence, with each variable explicitly listed as a fixed or random effect along with relevant interaction terms.**

*Thank you for highlighting this need for additional clarity. We have revised our ‘Statistical analyses’ section to address the questions raised in this comment and those raised above in this response to comments from reviewers document.*

*To summarize, we fit glmms to predict ghost crab burrow abundance (our response variable) in relation to date, temperature, habitat type, shell cover, and point type (****lines 183–194****). Our models also included a random effect of point ID to account for repeated measures (****line 208–209****). Because ghost crab burrow abundance is measured on a continuous scale [0, ∞) and because we detected evidence of overdispersion, we fit the glmms in the original submission with a negative binomial error distribution with a log link function (the default link function).*

*However, when we added the interactive effects to our model and checked assumptions, we found that the more complex model violated assumptions about zero-inflation. As a result, we fit our revised models as zero-inflated negative binomial models in which a zero-inflated component of the model predicts the probability of observing excess zeros, while a conditional component of the model predicts burrow counts conditional on the zero inflated component (****lines 202–210****). We fit glmms using the R package glmmTMB (****line 200*** *in ‘Methods’;* ***reference 39****, located on* ***line 529–532****).*

*As suggested, we have moved our discussion of the global model, defined as the model containing all covariates of interest, to a sentence that is separate from the discussion defining the type of model that we fit in the analysis. This sentence is located on* ***lines 210–212****: “Our global model contained the zero-inflated component for sampling date, and the conditional component included the random effect for sampling point, all five predictor variables as additive effects, and the two interactive effects.”*

**From the current analysis description, it is unclear whether you tested different distributions (e.g. negative binomial, poisson, or ordinary least squares) in the process of model selection.**

*Thank you for this question and opportunity for clarification. As mentioned above, our models changed as a result of adding interactive effects. We have made sure to include that we are using zero-inflated negative binomial models to account for overdispersion and zero-inflation in the data (****lines 201–202****).*  **Line 172— what are the a priori hypotheses that were tested in the candidate models? This is unclear based on the predictions outlined in the introduction and should be explicitly stated. “Considering what is known about ghost crab ecology” is a vague rationale for the choice of candidate models and additional text is needed here to explain and justify model construction.**

*Thank you for this suggestion and the opportunity to better clarify our hypotheses. We selected a set of predictor variables that we hypothesized would be important predictors of ghost crab activity, based on information in Lucrezi and Schlacher 2014 (****lines 183–194****). We describe each of those variables and why we selected them on* ***lines 183–194****. Based on this suggested revision, we’ve decided to include a table as supporting information (i.e., see* ***S1 Table****) that describes the* a priori *hypothesis associated with each candidate model included in our model set. For ease of review, we have copied that table here:*

*References used in this response:*

*Lucrezi S, Schlacher TA. Ecology of ghost crabs. In: Hughes RN, Hughes DJ, Smith IP, editors. Oceanography and marine biology: An annual review. Oxfordshire (UK): Taylor and Francis; 2014. pp. 201–256.*

**Results
Lines 182-183— is it not clear that the parentheticals contain the mean values, please include “mean” for each.**

*We’ve made the suggested revision throughout the result section, as needed (e.g., on* ***line 249****).* **Lines 188-191 – Please edit the parenthethicals containing statistical results to explicitly include SE and p-values , where appropriate for fixed effects.**

*Thank you. We’ve clarified that the parentheticals containing the beta coefficient values are the beta estimate plus/minus the SE (e.g.,* ***line 276****). Since we are using an information-theoretic approach, we cannot determine significance of fixed effects, as significance testing with p-values is incompatible with an information-theoretic framework (Burnham and Anderson 2004). Instead of using p-values to determine significance, we infer parameter importance of the top model using the 95% confidence intervals. When the CIs do not overlap (i.e., include) zero, we determine that the fixed effect is statistically informative.*

*In our earlier response, we outline the parameter betas, SEs, and confidence intervals. The CIs are also shown in Fig. 3 (if the error bars overlap or touch zero, we interpret as non-informative). As noted in an earlier response, we also outlined the informative parameters (i.e., 95% CIs with no overlap with zero) and noninformative parameters (i.e., 95% CIs overlap zero). For ease of review, we’ve included the table from that response here again:*

| ***Parameter*** | ***β*** | ***SE*** |  | ***95% confidence interval*** | | ***95% CI*** |
| --- | --- | --- | --- | --- | --- | --- |
|  |  |  |  | ***Lower limit*** | ***Upper limit*** | ***includes zero?*** |
| *Intercept* | *1.98* | *0.26* |  | *1.46* | *2.49* | *No* |
| *Beach habitat* | *-0.66* | *0.30* |  | *-1.26* | *-0.07* | *No* |
| *Berm habitat* | *-0.24* | *0.35* |  | *-0.92* | *0.44* | *Yes* |
| *Dune habitat* | *0.33* | *0.28* |  | *-0.21* | *0.88* | *Yes* |
| *Backdune flat habitat* | *0.67* | *0.33* |  | *0.03* | *1.32* | *No* |
| *Intertidal zone habitat* | *-0.72* | *0.52* |  | *-1.74* | *0.30* | *Yes* |
| *Overwash habitat* | *0.16* | *0.30* |  | *-0.43* | *0.75* | *Yes* |
| *American oystercatcher nest* | *-0.02* | *0.26* |  | *-0.51* | *0.49* | *Yes* |
| *Piping plover nest* | *0.58* | *0.51* |  | *-0.42* | *1.58* | *Yes* |
| *Sparse shell* | *0.27* | *0.19* |  | *-0.11* | *0.65* | *Yes* |
| *Heavy shell* | *-0.21* | *0.23* |  | *-0.67* | *0.25* | *Yes* |
| *Date* | *0.09* | *0.04* |  | *0.02* | *0.17* | *No* |
| *Temperature* | *0.27* | *0.04* |  | *0.19* | *0.35* | *No* |
| *American oystercatcher nest x sparse shell* | *-0.41* | *0.31* |  | *-1.02* | *0.20* | *Yes* |
| *Piping plover nest x sparse shell* | *-1.59* | *0.58* |  | *-2.73* | *-0.44* | *No* |
| *American oystercatcher nest x heavy shell* | *-0.68* | *0.42* |  | *-1.51* | *0.14* | *Yes* |
| *Piping plover nest x heavy shell* | *-2.22* | *1.04* |  | *-4.25* | *-0.19* | *No* |
| *Date x temperature* | *-0.06* | *0.04* |  | *-0.13* | *0.01* | *Yes* |
| *Zero-inflated intercept* | *-3.38* | *0.04* |  | *-4.17* | *-2.59* | *No* |
| *Zero-inflated date* | *-1.87* | *0.35* |  | *-2.54* | *-1.19* | *No* |

*References used in this response:*

*Burnham KP, Anderson DR. Multimodel inference: Understanding AIC and BIC in model selection. Socio Methods Res. 2004;33: 261–304. doi:10.1177/0049124104268644*

**Line 189— when examining Fig. 2, it does not appear that these are significant terms in model (date and temperature). Can the authors please explain?**

*We note that we use an information-theoretic framework, and thus, avoid the use of ‘significant’ which typically implies frequentist (p-value) testing (Burnham and Anderson 2004). We instead identify parameters as informative or uninformative, based on if their 95% confidence intervals overlap with zero (i.e., informative if the 95% CI does not overlap zero and uninformative if the 95% CI does overlap zero). We do provide additional information in our earlier responses above to both reviewers on our choice of these methods.*

*In our initial submission, Fig. 2 shows the date and temperature variables with small effect sizes and CIs (so small that the 95% CIs for these parameters are not clear on the figure). The 95% CIs for these parameters did not in fact overlap with zero, and thus we interpreted date and temperature to be informative parameters. However, we recognize that this was a significant design issue with the figure, and we have taken this into account in our revisions of the beta effects graph (formerly Fig. 2, now* ***Fig. 3****). The figure now has a more appropriate scale, and the sizing of the points and lines representing the beta estimates and 95% CIs (respectively) are adjusted to improve the figure’s readability.*

*Additionally, we are interpreting this comment to ask how our model can contain noninformative parameters (i.e., those whose 95% confidence intervals overlap with zero, as was the case for many habitat levels in the first submission). If we are incorrect on this interpretation, we welcome additional feedback so that we can fully address the critique and ensure our results are clearly and robustly presented.*

*In our original submission, we considered 17 candidate models that we evaluated in an information-theoretic framework. We identified the 17 candidate models based on* a priori *hypotheses (revised hypotheses presented in* ***S1 Table****, provided above in an earlier response). In our methodology, we first identify the model that ‘best’ fits the dataset using second-order Akaike’s information criterion (AIC_c_). While there are several ways authors may select the ‘best’ model from a list of plausible, candidate models, we identify the ‘best’ model as that with the greatest model weight (i.e., the relative likelihood the model fits the data and is bound by 0 [poor model fit of the data] and 1 [perfect model fit of the data]). We then considered models that were within 2 AIC_c_ units (i.e., ΔAIC_c_ ≤ 2.0) to be equally plausible options to the top-ranked model (Burnham and Anderson 2004). (Please note that in ‘Methods’, we revised our explanation of the model selection procedures on* ***lines 215–223*** *and of the parameter inference procedure on* ***lines 224–229****).*

*In our results, we considered one additional model (i.e., the model with all variables as additive effects only, and no interactive effects) to be nearly competitive (ΔAIC*_c_ *= 2.33), but because the interaction effects of multiple variables are only in one of the top models, we did not choose to model average and only report parameter estimates from the top-ranked model.*

*Although we have a robust sample size (829 observations from 101 total points), we cannot adequately consider every single possible model combination (~ 32 models based on models in our first submission which only considered five parameters as additive effects; ~ 128 models when we consider all possible combinations of the main effects and interactions considered in the revised submission) for all the covariates of interest, as doing so would be a form of data dredging and would be a serious statistical violation. Instead, we attempted to develop models representing* a priori *hypotheses as we note above, so as to avoid biologically implausible hypotheses. And while our top model in the first submission was well supported (model weight of top model = 0.94, see Johnson and Omland 2004), some parameters within the model may not be statistically informative. Further editing the top model to remove the uninformative parameters would effectively change the model list and would be a form of ‘data fishing,’ in an AIC framework. Because authors must balance data limitations and ‘data fishing/dredging,’ it’s not atypical to see top models with uninformative parameters, as is the case for some recent publications by our coauthors (e.g., Wails et al. 2024).*

*In our revised manuscript, our top ranked model was the global model. The table that we provided in a prior response indicated which parameters had confidence intervals that overlapped with zero and were thus uninformative. In this particular instance, it could be that piping plover nests (additive effect only), for example, have an effect on crab burrow abundance but we lack the sufficient sample size to detect such an effect. However, the simplest conclusion we’re able to make is that there is no effect of piping plover nests (additive effect only) on burrow abundance in this study.*

*References used in this response (in order of mention):*

*Burnham KP, Anderson DR. Multimodel inference: Understanding AIC and BIC in model selection. Socio Methods Res. 2004;33: 261–304. doi:10.1177/0049124104268644*

*Johnson JB, Omland KS. Model selection in ecology and evolution. Trends Ecol Evol. 2004;19: 101–108. doi:10.1016/j.tree.2003.10.013*

*Wails, CN, Helmke, CC, Black, KM, Ramirez-Barrios, R, Karpanty, SM, Catlin, DH, Fraser, JD. Epidemiology of sarcoptic mange in a geographically constrained insular red fox population 2024. Epidemiology of sarcoptic mange in a geographically constrained insular red fox population. Parasites and Vectors.* *2024;17, 248. https://doi.org/10.1186/s13071-024-06330-5*

**Line 190— if habitat was included as a fixed effect in the model, how are you reporting statistical results for levels within habitat? This is not clear in the analysis methods, and if post hoc tests with multiple comparisons were made then this should be explicit.**

*Thank you for the opportunity to clarify. As we note above, we use glmms to fit our data. Several fixed effects are included as numeric covariates (e.g., date, temperature) and other fixed effects are categorical (e.g., shell cover, habitat type, point type).*

*When analyzing with regression methods, like glmms, beta coefficients are fit differently for continuous and categorical variables (Zar 2010). For a categorical variable, a reference level is chosen (typically a ‘control’ variable) and its beta and SE are set to zero (Zar 2010). For all other levels in the categorical variable, beta is calculated relative to the reference level (Zar 2010). Thus, in the case of our point type variable, we had random locations (no nest; used as the reference level with beta and SE set to 0), piping plover nests, and American oystercatcher nests. The beta values for piping plover and American oystercatcher nests are relative to the reference level. The same is true for habitat: we selected island backbarrier habitat as a reference level, and all other habitat types (e.g., levels) are relative to the reference level. This analysis is not a type of post-hoc test, but rather a standard regression method to calculate beta coefficients of categorical variables (Zar 2010); hence, there is no post-hoc test.*

*As we have noted earlier, we use an information-theoretic approach that is incompatible with p-values (Burnham and Anderson 2004). Instead, we use AIC to identify the model (vs backward model selection where one would develop a maximal model and remove non-significant fixed effects using Wald’s chi or a similar appropriate statistic). We then explore the effects within the ‘best’ AIC-ranked model using the beta coefficients. In a frequentist approach, one could view the beta coefficients and corresponding standard errors for parameters, which would be identical to what we present here for categorical variables. In an AIC framework, we use confidence intervals to infer importance of coefficients (clarified on* ***lines 227–229****), whereas one would likely use Z-statistics and p-values to identify significance of coefficients.*

*To summarize, we did not conduct a post-hoc test, and our beta coefficients reflect standard methods of regression analyses for categorical data. In our revised methods, we do not note any post-hoc tests. We likewise clarified in the caption of Fig. 3 that some of the fixed effects are categorical in nature and thus include reference levels (****lines 284–291****).*

*References in this response (in order of mention):*

*Zar JH. Biostatistical Analysis, 5th ed. Upper Saddle River (NJ): Pearson; 2010.*

*Burnham KP, Anderson DR. Multimodel inference: Understanding AIC and BIC in model selection. Socio Methods Res. 2004;33: 261–304. doi:10.1177/0049124104268644*

**Discussion
Line 248— delete “However”**

*Thank you. We have made the suggested revision, which is now on* ***line 344****.*

**Line 271— change “may need to” to “should”**

*Thank you. We have made the suggested revision, which is now on* ***line 368****.*

**Line 274— authors should be clear what they mean by “future research” directions. For example, it seems like it would be helpful to have data that could potentially help capture phenological shifts in predation risk. The authors should also consider including a final conclusion sentence (or two) that highlights the strength of their contribution and how this study can provide a basis for these future research in this area.**

*Thank you! We agree that these future directions would be excellent for further study. We’ve modified our concluding paragraph (starting on line 372) to be more specific about future directions: “Further research is needed to understand how habitat characteristics and environmental factors influence spatiotemporal ghost crab activity and overlap with shorebird breeding phenology across sites. Understanding microhabitat characteristics selected by both ghost crabs and nesting shorebirds will help to identify practical and effective strategies for managing the threat of ghost crabs to shorebird reproductive success. For example, shell placement could promote successful shorebird nesting by limiting ghost crab burrow construction and reducing ghost crab activity at nest sites. Additionally, further investigation into the potential for a finer-scale association between temperature, date, and crab burrow abundance and for a phenological shift in either ghost crab activity or shorebird reproduction may help to assess if the ghost crab predation risk to shorebird eggs and chicks is evolving in response to climate change.”*

**Line 275—change to “spatiotemporal ghost crab activity” as the crab is the noun in this sentence.**

*Thank you. We have made the suggested revision, now on* ***line 373****.*  **Figures and Tables
Table 1— It is not necessary to identify all levels for each variable of the model in the caption, but the variables should be clearly listed as fixed factors. If not explicitly including the random effect on each line, the caption should read “All models included a random effect of sampling point. Additionally, the code is not available so it is not possible to check whether the random effect was coded properly.**

*Thank you for the suggestions. We have revised the caption for Table 1 (now on* ***lines 259–264****) to clarify that only the fixed effects are listed, and that all models also include a random effect for sampling point.*

*We fit glmms using the R package glmmTMB (****line 200*** *in ‘Methods’;* ***reference 39****, located on* ***line 529–532****). Our global model was encoded encoded according to what is specified for that package, as:*

*glmmTMB(burrow.count ~ (1| point.id) + habitat + point.type+ shell.cover + date + temp +*

*point.type* × *shell.cover + date* × *temp,*

*ziformula = ~ date,*

*family = nbinom2, data = count)*

*Where “burrow.count ~ (1| point.id) + habitat + point.type+ shell.cover + date + temp + point.type × shell.cover + date × temp” was the conditional model component, and “ziformula = ~ date” was the zero-inflated model component (as per ref 39). All candidate models included the random effect for point ID (1 | point.id) in the conditional model component, and all candidate models included the zero-inflated model component. Fixed effects within the conditional model component changed depending on the candidate model, as outlined in Table S1.*  **The manuscript has minimal figures. While I think the present figures/tables are effective, I suggest that the authors consider including an image figure to show nest sites and random point sites, highlighting the burrows that were counted. This could be included in the main text given the current low figure count, or included as an appendix.**

*Thank you for this thoughtful suggestion! We’ve included the suggested figure (now* ***Fig. 2****) in the manuscript as we think that it will provide a useful visual representation of our survey methods.*

**6. PLOS authors have the option to publish the peer review history of their article (**[**what does this mean?**](https://nam04.safelinks.protection.outlook.com/?url=https%3A%2F%2Fjournals.plos.org%2Fplosone%2Fs%2Feditorial-and-peer-review-process%23loc-peer-review-history&data=05%7C02%7Ckarpanty%40vt.edu%7Ca473dabafc3f44133d6008dc856f14e8%7C6095688410ad40fa863d4f32c1e3a37a%7C0%7C0%7C638531961533269352%7CUnknown%7CTWFpbGZsb3d8eyJWIjoiMC4wLjAwMDAiLCJQIjoiV2luMzIiLCJBTiI6Ik1haWwiLCJXVCI6Mn0%3D%7C0%7C%7C%7C&sdata=ntDtw2SgdDrWG5mf314Q2Y2rwlOv913%2B670z6lr%2BV5Y%3D&reserved=0)**). If published, this will include your full peer review and any attached files.


If you choose “no”, your identity will remain anonymous but your review may still be made public.


Do you want your identity to be public for this peer review? For information about this choice, including consent withdrawal, please see our** [**Privacy Policy**](https://nam04.safelinks.protection.outlook.com/?url=https%3A%2F%2Fwww.plos.org%2Fprivacy-policy&data=05%7C02%7Ckarpanty%40vt.edu%7Ca473dabafc3f44133d6008dc856f14e8%7C6095688410ad40fa863d4f32c1e3a37a%7C0%7C0%7C638531961533282955%7CUnknown%7CTWFpbGZsb3d8eyJWIjoiMC4wLjAwMDAiLCJQIjoiV2luMzIiLCJBTiI6Ik1haWwiLCJXVCI6Mn0%3D%7C0%7C%7C%7C&sdata=LURurrzyEGpl5dXORqO8AWrq5gJ0qo58VIFRMMG0VqU%3D&reserved=0)**.**

**Reviewer #1: No**

**Reviewer #2: No**
